# Preconcentration and Determination of Mefenamic Acid in Pharmaceutical and Biological Fluid Samples by Polymer-grafted Silica Gel Solid-phase Extraction Following High Performance Liquid Chromatography

**Published:** 2015

**Authors:** Hayedeh Bagheri Sadeghi, Homayon Ahmad Panahi, Mahsa Mahabadi, Elham Moniri

**Affiliations:** a *Department of Chemistry, Central Tehran Branch, Islamic Azad University, Tehran, Iran.*; b *Department of Chemistry, Varamin (Pishva) Branch, Islamic Azad University, Varamin, Iran.*

**Keywords:** Mefenamic acid, Solid phase extraction, High performance liquid chromatography, Pharmaceutical sample, Biological sample

## Abstract

Mefenamic acid is a nonsteroidal anti-inflammatory drug (NSAID) that has analgesic, anti-infammatory and antipyretic actions. It is used to relieve mild to moderate pains. Solid-phase extraction of mefenamic acid by a polymer grafted to silica gel is reported. Poly allyl glycidyl ether/iminodiacetic acid-co-N, N-dimethylacrylamide was synthesized and grafted to silica gel and was used as an adsorbent for extraction of trace mefenamic acid in pharmaceutical and biological samples. Different factors affecting the extraction method were investigated and optimum conditions were obtained. The optimum pH value for sorption of mefenamic acid was 4.0. The sorption capacity of grafted adsorbent was 7.0 mg/g. The best eluent solvent was found to be trifluoroacetic acid-acetic acid in methanol with a recovery of 99.6%. The equilibrium adsorption data of mefenamic acid by grafted silica gel was analyzed by Langmuir model. The conformation of obtained data to Langmuir isotherm model reveals the homogeneous binding sites of grafted silica gel surface. Kinetic study of the mefenamic acid sorption by grafted silica gel indicates the good accessibility of the active sites in the grafted polymer. The sorption rate of the investigated mefenamic acid on the grafted silica gel was less than 5 min. This novel synthesized adsorbent can be successfully applied for the extraction of trace mefenamic acid in human plasma, urine and pharmaceutical samples.

## Introduction

Mefenamic acid is a non-steroidal anti-inflammatory drug used to treat pain, including menstrual pain. It is typically prescribed for oral administration. It is usually available in 100 mg, 250 mg, 500 mg tablets or 250 mg capsules. Mefenamic acid can cause side effects such as headache, vomiting, diarrea, haematuria, hematemesis. These symptoms appear while taking this medication. Recently, non-steroidal anti-inflammatory drugs are broadly used for treatment of patients with rheumatoid arthritis (1, 2). These compounds non selectively inhibit the two isoforms of the cyclooxygenase (COX-1 and COX-2) and so prevent the metabolism of cellular arachidonic acid (AA) and the upregulation of prostaglandin formation, which in other respects lead to an increase of vascular permeability, edema, hyperalgesia, pyrexia and inflammation (3,4). 

**Scheme 1:**
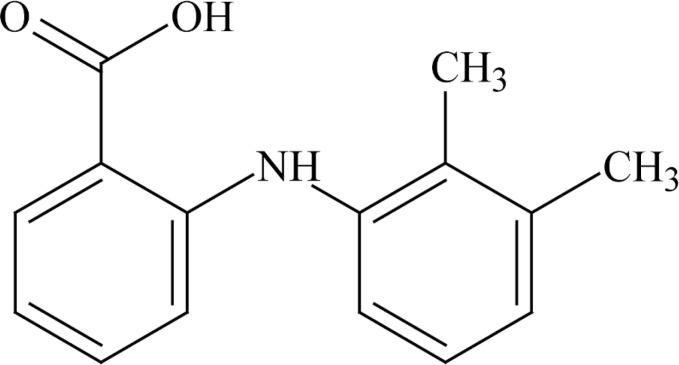
Structure of mefenamic acid

The non-steroidal anti-inflammatory drugs are divided into eight groups according to their structures. Amongst these groups anthranillic acid derivatives are of great importance. Mefenamic acid (see scheme 1 for molecular structure), 2-[2, 3-dimethylphenyl) amino] benzoic acid, is an anthranillic acid derivative with anti-inflammatory properties (5, 6). 

Several analytical methods for determination of mefenamic acid in biological and pharmaceutical samples have already been developed and reported. These analytical methods include electrochemical analysis (7-10), spectrophotometry (11-13), capillary electrophoresis (14, 15), chromatography (16-18), atomic absorption spectrometry (19), fluorescence spectrometry (20), nuclear magnetic resonance spectroscopy (21), flow injection analysis (22). 

To promote the sensitivity and selectivity of an analytical method, development and application of a practical sample preparation procedure prior to quantification is of great importance. Solid -phase extraction is a very common method for rapid sample preparation. The versatility of this technique allows us to use it for many purposes, such as preconcentration, purification, separation and trace enrichment. In the last few years, there are many publications about the use of solid -phase extraction in pharmaceutical products (23, 24). Amongst the solid -phase extraction sorbents, silica gel plays an important role, since it does not swell or strain and has a good mechanical strength. The surface of silica gel particles is heterogeneous, with a variety of silanol groups which are excellent groups for modification reactions. Surface modification by grafting polymer chain to solid substrate is a useful method for creation of materials which possess specific surface and structural properties which can be used for preconcentration of trace analytes. 

In this research, the performance of a novel synthetic support in adsorption of mefenamic acid is evaluated. To achieve this goal, free-radical graft co-polymerization of N, N-dimethylacrylamide and a designed monomer, Allyl glycidyl ether/iminodiacetic acid (AGE/IDA) onto modified silica with (3-mercaptopropyl) trimethoxy silane was performed. The interactions between the mefenamic acid and the active sites in the polymer are frequently hydrogen bonding and have reversible character for sorption and desorption phenomena. This novel polymer grafted silica gel in batch-wise mode, was used for the pretreatment of trace mefenamic acid in human urine and plasma prior to the determination of concentration of mefenamic acid by high performance liquid chromatography (HPLC). 

## Experimental

*Reagents*

Silica Gel, Iminodiacetic acid (IDA), 2,2´-Azobis(2-methyl propionitrile), 3-mercapto propyltrimethoxysilane, 1,4 Dioxane, acetone, ethanol, acetic acid (AA), sodium acetate, Trifluoroacetic acid (TFA) were all obtained from Merck (Darmstadt, Germany) and N,N-Dimethylacrylamide (DMAA) was purchased from Sigma-Aldrich (St.Louis, Mo, 63103 United States). Allyl glycidyl ether (AGE) was purchased from Fluka Chemicals. Acetonitrile and tetrahydrofuran were of HPLC grade and used without any further purification. 

The stock solution of mefenamic acid was prepared by dissolving appropriate amount in deionized water. To adjust the pH of the solutions, acetic acid-acetate buffer or phosphate buffer were used wherever suitable. 

*Instruments*

Chromatographic separations were carried out on a Shimadzu HPLC, 1200 series, equipped with UV/Vis detector. Separations were carried out on C18 column (150×460 mm), using 50 mM solution of monobasic ammonium phosphate, and adjusted with 3M ammonium hydroxide to a pH of 5.0 as the buffer solution at a flow- rate of 1 mL per minute. The effluent from the column was monitored at 280 nm. The pH measurements were made by a Metrohm model 744 pH meter (Zolingen, Switzerland). Infrared spectra were recorded on a Jasco Fourier Transform infrared spectrometer (FTIR-410, Jasco Inc.Easton, Maryland). The UV/Vis spectra were obtained by JascoV-530 UV/Vis spectrometer (Jasco Inc.Easton, Maryland). 

*Preparation of adsorbent*

Synthesis of poly [1-N,N-(bis-carboxymethyl)amino-3-allylglycerol-co-dimethylacrylamide](poly(AGE/IDA-co-DMAA) grafted onto silica gel (PAID-GSG)has already been reported by H.A.Panahi, *et al.* (24,25). Briefly, the synthesis of PAID-GSG was done in four stages:

1) Synthesis of the functional monomer, allylglycerol/ Iminodiacetic acid (AGE/IDA) 

2) Silica gel activation using hydrochloric acid 

3) Modification of activated silica gel with 3-mercaptopropyltrimethoxysilane 

4) Graft copolymerization of AGE/IDA and dimethylacrylamide (DMAA) onto modified silica particles

**Scheme 2:**
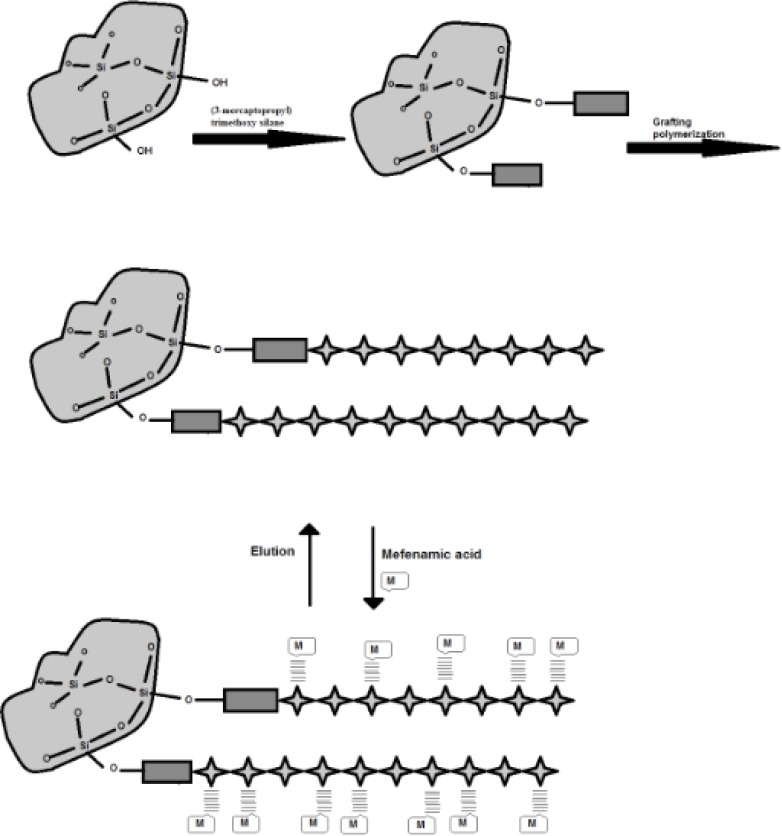
Schematic presentation of PAID-GSG synthesis process

**Figure 1:**
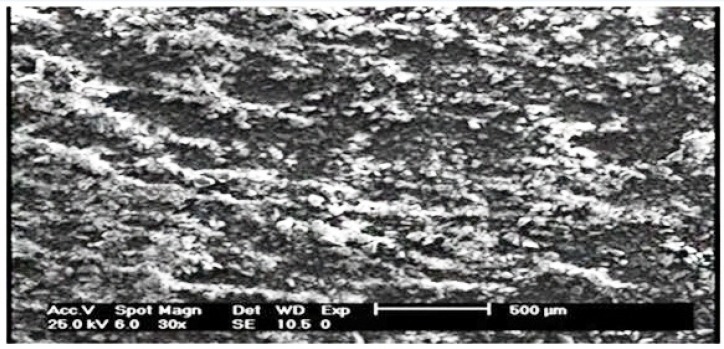
Scanning electron microscopy (SEM) photograph of the PAID-GSG

The methodology used to synthesize PAID-GSG is summarized in (Scheme 2). The PAID-GSG was characterized by FT-IR, elemental analysis, and thermogravimetric analysis and scanning electron microscopy (SEM).The SEM photograph of synthesized adsorbent is given in (Figure 1).

*Method procedure*

The degree of mefenamic acid sorption at different pH values was determined by the batch equilibration technique. To study this effect, a set of 10 µg/mL solutions (25 mL) containing 0.02 g of PAID-GSG with different pH values ranging from 3 to 7 were vortexed for 20 min and then centrifugated for 10 min and content of mefenamic acid was measured in supernatant liquid at 280 nm by UV-Vis spectrometry.

In method procedure for best elution solvent, a set of micro-tubes each containing 1 mL of 2 µg/mL solution of mefenamic acid and 0.01 g PAID-GSG was vortexed for 20 min, centrifugated for 10 min and subsequently the supernatant was decanted. Then 1 mL of elution solvent was added to the polymer, sonicated at 55 ºC for 1 h, then vortexed for 15 min and finally centrifugated. The concentration of mefenamic acid in eluent was determined by injection to HPLC column.

*Isotherm studies*

Isotherm studies were performed by adding 0.01 g of PAID-GSG to a series of micro-test tubes filled with 1 mL diluted solutions of mefenamic acid (10-60 μg/mL) at pH 4. The micro-tubes were vortexed for 20 min. The amount of mefenamic acid at equilibrium q_e_ (mg/g) on PAID-GSG was calculated from the following equation:

q_e_= (C_0_-C_e_) V/W                      Equation (1)

Where C_0_ and C_e_ (mg/L) are initial and equilibrium concentration of the mefenamic acid, respectively, V (L) is the volume of the solution and W (g) is the mass of the PAID-GSG. 

## Results and Discussion

*Optimization of parameters*

*Effect of pH*

**Figure 2:**
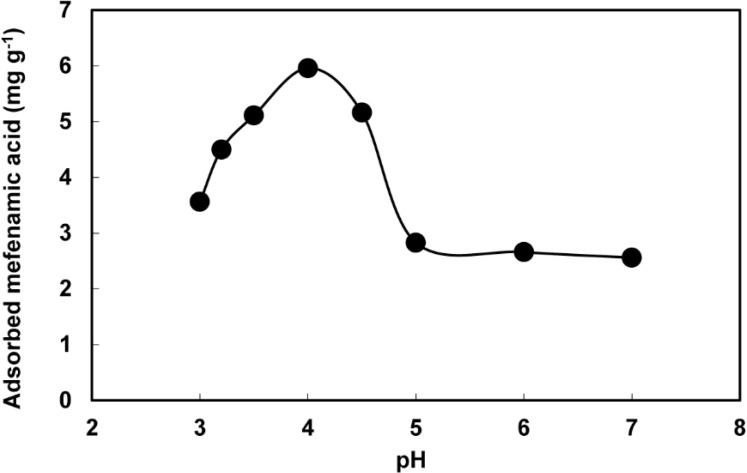
Effect of pH on sorption of mefenamic acid onto PAID-GSG

The experimental results of mefenamic acid sorption as a function of pH are shown in Figure 2. The best extraction was found to be at pH 4.0. The solubility of mefenamic acid with carboxylic acid group is minimum at acidic pH, therefore higher extraction is achieved. pH values more than 7 were not considered because of solubility of silica gel in alkaline pH. 

*Effect of adsorption time*

**Figure 3:**
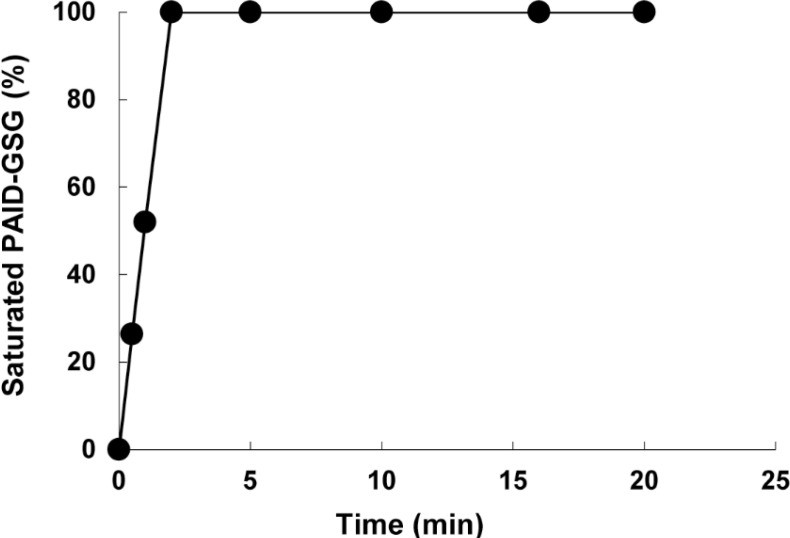
Kinetics of mefenamic acid sorption onto PAID-GSG

To investigate the time required for optimum adsorption of mefenamic acid onto polymer, 0.02 g of polymer was shaken with 10 mL of solution containing 10 mg/L mefenamic acid at pH 4 and different length of time (0.5-20 min). After centrifugation, the concentration of mefenamic acid in supernatant solution was determined by UV-Vis spectrometry. As shown in (Figure 3) about 2 min was required to reach 100% sorption saturation, but to ensure that sorption is complete 20 min was chosen as the optimum adsorption time.

*Effect of different eluent solvents on the recovery*

The effect of various eluent on the extraction recovery of mefenamic acid was also investigated. The eluents used were methanol, AA and TFA. Presence of AA and TFA in eluent improved the recovery (26-28). Amongst the eluents listed in Table 1, methanol containing AA (0.2%) and TFA (1%) shows the best result and can be used for optimum removal of the mefenamic acid from PAID-GSG.

**Table 1:** Recovery evaluation of Mefenamic acid in different eluents

**No**	**Eluent**	**Recovery (%)**
1	MeOH^a^/TFA^b^ (2%)	55.8
2	MeOH/TFA (5%)	48.6
3	MeOH/AA^c^ (0.1%)/TFA (1%)	70.0
4	MeOH/AA (0.1%)/TFA (2%)	40.0
5	MeOH/AA (0.2%)/TFA (1%)	99.6
6	MeOH/AA (1%)/TFA (1%)	46.5
7	MeOH/AA (2%)/TFA (1%)	10.8

a )methanol

b )trifluoroacetic acid

c )acetic acid

*Adsorption isotherm*

The kinetic sorption of mefenamic acid at pH 4 is shown in (Figure 3). As shown 2 min was required to reach 100% sorption saturation. The profile of mefenamic acid uptake on the sorbent reflects the good accessibility of binding sites in the PAID-GSG. Maximum sorption capacity can be defined by Langmuir isotherm which is valid for monolayer sorption on PAID-GSG surface with number of identical active sites. In Langmuir isotherm it is assumed that the energies of sorption on the PAID-GSG surface are uniform and no transmigration of mefenamic acid occurs in the plane of the surface (29). The Langmuir equation can be expressed:

C_e_/q_e_= (1/q_max_.K_L_) + (C_e_ /q_max_)                      Equation(2)

Where q_max_ and K_L_ are the maximum mefenamic acid sorption capacity corresponding to complete monolayer coverage on the PAID-GSG surface and the Langmuir constant, respectively. 

**Figure 4:**
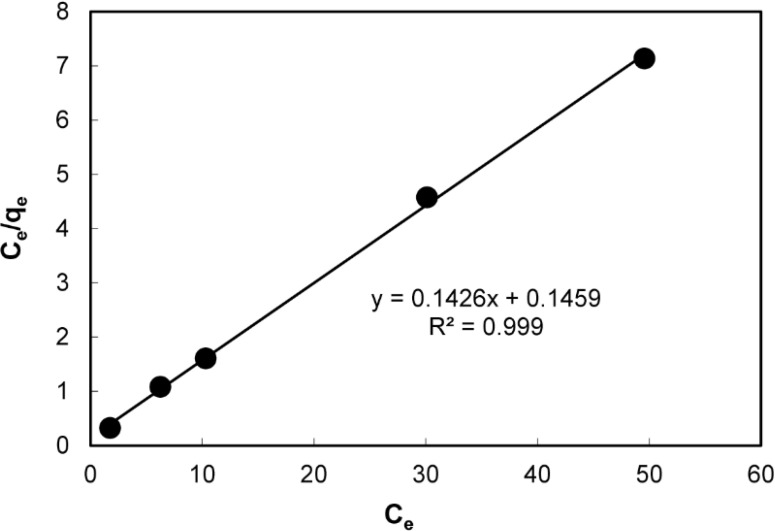
Langmuir isotherm for mefenamic acid adsorption onto PAID-GSG at 20 °C.

The linear regression equation in (Figure 4) suggested that the homogeneous sites for mefenamic acid were formed in the PAID-GSG .From the slope (1/q_max_) and intercept (1/q_max_.K_L_) of this line, K_L_ and q_max_ were calculated to be 0.98 L/mg and 7.0 mg/g, respectively.

The essential characteristic of the Langmuir model is expressed with R_L_ :

R_L_ =1 / (1+K_L_.C_0_)                       Equation (3)

The calculated R_L_ value was 0.017 that is in the range of 0-1 that indicates the adsorption nature is favourable (0<R_L_<1) (29-30). 

*Determination of mefenamic acid in capsules*

The proposed SPE-HPLC procedure was successfully applied for determination of mefenamic acid in capsules. Capsules of mefenamic acid (20 capsules) bought from local pharmacy (250 mg capsules from Amin pharmaceutical company) were weighed accurately and powdered using a mortar and pestle. A portion of the powder equal to the weight of one capsule was dissolved in methanol and was shaken for 2 hours and then it was sonicated for 15 minutes in order to aid dissolution and then filtered. The filtrate was diluted by water and a 2 µg/mL solution was prepared. The method procedure was performed on this sample for extraction and determination of mefenamic acid content. The concentration of mefenamic acid was obtained from calibration curve. Results are shown in Table 2. The results shown in this table indicate the applicability of this procedure with high recovery. 

**Table 2:** Determination of Mefenamic acid in different samples

**Sample**	**Concentration of mefenamic acid(µg/mL)**	**Added)** **(µg/mL)**	**Found** * **(µg/mL)**	**Recovery (%) **
Plasma	_____	2.00	1.70±0.28	85.0
Urine	_____	2.00	1.86 ±0.11	95.0
Capsule**	2.00	_____	1.80±0.19	90.0

* For three determinations (n=3)

** 250 mg capsules from Amin pharmaceutical company

*Determination of mefenamic acid in human plasma and urine*

Human blood was collected from thoroughly controlled voluntary blood donors. Each blood-containing unit was separately controlled and found negative for Hbs antigen and HIV I, II and hepatitis C antibodies. The red blood cells were separated from the plasma by centrifugation at 4500×g for 30 min at room temperature, then filtered and frozen at -20 ºC. Before use, the plasma was thawed for 1 h at 37 ºC. In order to reduce the matrix effect, the plasma was deproteinized by mixing it with equal volume of acetonitrile (31), vortexed for 3 min, centrifugated for 5 min at 1500×g, filtered (24 mµ) spiked with mefenamic acid. A spiked urine sample was prepared directly from urine sample. Spiked samples of plasma and urine were subjected to the proposed extraction procedure and injected to HPLC column. Results are given in Table 2, which demonstrates the reasonability and reliability of the proposed extraction procedure.

*Analytical performance of the method*

Six replicate determination of a 2 µg/mL solution of mefenamic acid gave a relative standard deviation (RSD %) of 0.65 %. The limit of detection (LOD)corresponding to three times the blank standard deviation divided by slope of calibration curve was found to be 0.46 µg/L. The limit of quantification (LOQ) corresponding to ten times the blank standard deviation was found to be 1.5 µg/L. The concentration of mefenamic acid were obtained from calibration curve with regression equation of A=22.88C+24.278, in which A is the area under the peak and C is the concentration of mefenamic acid (µg/mL) with correlation of coefficient (R^2^ =0.9919). 

## Conclusions

A novel polymer grafted to silica gel (PAID-GSG) was synthesized and used for solid phase extraction of mefenamic acid followed by HPLC determination. The results indicate that the suggested method has some advantages such as little solvent consumption, high chemical stability, simplicity, extraction efficiency, less time consuming compared to other adsorbents. The sorption rate of the investigated mefenamic acid on the PAID-GSG was excellent (about 2 min). The PAID-GSG can be applied to the extraction of trace mefenamic acid in human plasma, urine and pharmaceutical samples with satisfactory results.
